# Trends in antimicrobial resistance in bloodstream infection isolates at a large urban hospital in Malawi (1998–2016): a surveillance study

**DOI:** 10.1016/S1473-3099(17)30394-8

**Published:** 2017-10

**Authors:** Patrick Musicha, Jennifer E Cornick, Naor Bar-Zeev, Neil French, Clemens Masesa, Brigitte Denis, Neil Kennedy, Jane Mallewa, Melita A Gordon, Chisomo L Msefula, Robert S Heyderman, Dean B Everett, Nicholas A Feasey

**Affiliations:** a Malawi-Liverpool-Wellcome Trust Clinical Research Programme, Blantyre, Malawi; b College of Medicine, University of Malawi, Blantyre, Malawi; c Queen's University, Belfast, UK; d Institute of Infection and Global Health, University of Liverpool, Liverpool, UK; e Division of Infection and Immunity, University College London, London, UK; f Liverpool School of Tropical Medicine, Liverpool, UK

## Abstract

**Background:**

Bacterial bloodstream infection is a common cause of morbidity and mortality in sub-Saharan Africa, yet few facilities are able to maintain long-term surveillance. The Malawi-Liverpool-Wellcome Trust Clinical Research Programme has done sentinel surveillance of bacteraemia since 1998. We report long-term trends in bloodstream infection and antimicrobial resistance from this surveillance.

**Methods:**

In this surveillance study, we analysed blood cultures that were routinely taken from adult and paediatric patients with fever or suspicion of sepsis admitted to Queen Elizabeth Central Hospital, Blantyre, Malawi from 1998 to 2016. The hospital served an urban population of 920 000 in 2016, with 1000 beds, although occupancy often exceeds capacity. The hospital admits about 10 000 adults and 30 000 children each year. Antimicrobial susceptibility tests were done by the disc diffusion method according to British Society of Antimicrobial Chemotherapy guidelines. We used the Cochran-Armitage test for trend to examine trends in rates of antimicrobial resistance, and negative binomial regression to examine trends in icidence of bloodstream infection over time.

**Findings:**

Between Jan 1, 1998, and Dec 31, 2016, we isolated 29 183 pathogens from 194 539 blood cultures. Pathogen detection decreased significantly from 327·1/100 000 in 1998 to 120·2/100 000 in 2016 (p<0·0001). 13 366 (51·1%) of 26 174 bacterial isolates were resistant to the Malawian first-line antibiotics amoxicillin or penicillin, chloramphenicol, and co-trimoxazole; 68·3% of Gram-negative and 6·6% of Gram-positive pathogens. The proportions of non-*Salmonella* Enterobacteriaceae with extended spectrum beta-lactamase (ESBL) or fluoroquinolone resistance rose significantly after 2003 to 61·9% in 2016 (p<0·0001). Between 2003 and 2016, ESBL resistance rose from 0·7% to 30·3% in *Escherichia coli*, from 11·8% to 90·5% in *Klebsiella* spp and from 30·4% to 71·9% in other Enterobacteriaceae. Similarly, resistance to ciprofloxacin rose from 2·5% to 31·1% in *E coli*, from 1·7% to 70·2% in *Klebsiella* spp and from 5·9% to 68·8% in other Enterobacteriaceae. By contrast, more than 92·0% of common Gram-positive pathogens remain susceptible to either penicillin or chloramphenicol. Meticillin-resistant *Staphylococcus aureus* (MRSA) was first reported in 1998 at 7·7% and represented 18·4% of *S aureus* isolates in 2016.

**Interpretation:**

The rapid expansion of ESBL and fluoroquinolone resistance among common Gram-negative pathogens, and the emergence of MRSA, highlight the growing challenge of bloodstream infections that are effectively impossible to treat in this resource-limited setting.

**Funding:**

Wellcome Trust, H3ABionet, Southern Africa Consortium for Research Excellence (SACORE).

## Introduction

Bloodstream infection is a leading cause of morbidity and mortality in both adults and children in sub-Saharan Africa.^1^ In this region, the high burden of bacterial bloodstream infection has been strongly associated with the high prevalence of HIV, malaria, and malnutrition.^1^, ^2^, ^3^, ^4^ The clinical effect of bloodstream infections in sub-Saharan Africa is exacerbated by the inadequacy of diagnostic facilities, precluding both timely diagnosis of severe bacterial infection and implementation of appropriate antimicrobial therapy.^5^

Since 1998, sentinel bacteraemia surveillance has been done at Queen Elizabeth Central Hospital (QECH), Blantyre, Malawi, a setting with a high prevalence of HIV, malaria, and malnutrition.^6^ Blantyre is one of two principal cities in Malawi and the population of its urban and peri-urban rural areas expanded rapidly during the study period. QECH is one of the largest government hospitals in Malawi and is the only public hospital providing free medical care to Blantyre city, serving an urban population of 920 000 in 2016. The hospital has about 1000 beds, although occupancy frequently exceeds capacity. The hospital admits about 10 000 adults (aged 16 years or older) and 30 000 children (aged younger than 16 years) per year. From 1998 to 2015, sepsis was treated either with chloramphenicol and benzylpenicillin, or with ceftriaxone, which was introduced in Malawi in 2004. Ceftriaxone was not widely available in the city or district of Blantyre outside of QECH; however, it was extensively used as a first-line agent at QECH and was given to 90·0% of febrile adult patients admitted to QECH in one study in 2009–10.^7^ During this same period, antiretroviral therapy (ART) programmes, malaria control interventions, and improvements in food security and community management of malnutrition were rolled out. HIV prevalence in Blantyre decreased from 22·3% in 2004, to 17·6% in 2016, while enrolment on the ART programme in Malawi increased from 4000 (2·3% of those in need of ART) in 2004, to more than 530 000 (67·0% those in need of ART) in 2014.^8^, ^9^, ^10^, ^11^ Conjugate vaccines have been introduced against *Haemophilus influenzae* type b (in 2002) and pneumococcus (in 2011).^12^, ^13^, ^14^ Over the period of bloodstream infection surveillance, there were also considerable reductions in mortality among children younger than 5 years and among HIV-infected adults.^7^, ^15^, ^16^

**Research in context**

**Evidence before this study**

Long-term surveillance data describing bloodstream infection and antimicrobial resistance in sub-Saharan Africa are scarce. A systematic review and meta-analysis of community-acquired bloodstream infection in Africa by Reddy and colleagues identified only 22 studies over a period of more than 20 years (up to June, 2009) and most (13) focused on children. Only one study from Egypt and none from sub-Saharan Africa reported long-term surveillance data for both children and adults, and only 13 studies reported antimicrobial susceptibility data. A search of PubMed (using the terms: “Africa” AND “community acquired” AND [“bacteraemia” OR “sepsis”]) for July, 2009–June, 2016, revealed two large bacteraemia datasets; one from South Africa containing 17 001 blood culture results from a 6-year period and the other from Mozambique containing a further 19 896 blood culture results from a 5-year period. These studies reported trends in community-acquired bloodstream infection and antimicrobial resistance in all pathogens, but only for paediatric patients. We found no studies detailing longitudinal passive bloodstream infection surveillance from both adults and children.

**Added value of this study**

Like many sub-Saharan African countries, Malawi is under considerable pressure from poverty, undernutrition, urbanisation, malaria, and HIV. Blantyre is a major African city, grappling with the same issues of rapid expansion in population size with insufficient access to water and sanitation as elsewhere on the continent. We present the largest bacteraemia and antimicrobial resistance surveillance dataset yet collected from sub-Saharan Africa and describe trends in bloodstream infection in both adults and children presenting to a major urban teaching hospital in Malawi over a 19-year period. Our study reveals a decline in the incidence of bloodstream infection caused by all pathogens except *Salmonella* Typhi. This decrease has occurred concurrently with several major public health interventions, including the extensive roll-out of both antiretroviral therapy and malaria control interventions, improvements in food security and the community management of malnutrition, and the introduction of *Haemophilus influenzae* type b and pneumococcal conjugate vaccines. This good news is tempered by the emergence and rapid expansion of drug-resistant pathogens, including cephalosporin-resistant and fluoroquinolone-resistant Enterobacteriaceae, penicillin-resistant *Streptococcus pneumoniae* and meticillin-resistant *Staphylococcus aureus*.

**Implications of all the available evidence**

Although there has been a marked decrease in community-acquired bacterial bloodstream infection in Malawi, more pathogens are becoming effectively untreatable because of their resistance to locally available antimicrobial agents. As alternative agents such as carbapenems are expensive and currently unavailable in this resource-limited setting, actions to help mitigate further spread of resistance to the available antimicrobials are urgently needed.

Non-typhoidal salmonellae, *Salmonella enterica* serotype Typhi, and *Streptococcus pneumoniae* were previously identified as leading causes of bloodstream infection.^6^, ^17^, ^18^ Widespread multidrug-resistant non-typhoidal salmonella necessitated the introduction and increasingly extensive use of ciprofloxacin (in 2002) and ceftriaxone (in 2004) for the management of sepsis.^17^, ^19^ Extended spectrum beta-lactamase (ESBL)-producing and fluoroquinolone-resistant Enterobacteriaceae have been reported in different settings worldwide where cephalosporins and fluoroquinolones have been in use,^20^, ^21^, ^22^, ^23^, ^24^ including Blantyre, where ESBL-producing and fluoroquinolone-resistant *Escherichia coli*, klebsiella, and salmonella isolates have been identified previously;^7^, ^25^, ^26^ however, the full burden of ESBL and fluoroquinolone resistance among Gram-negative pathogens in this setting has yet to be described.

Among Gram-positive pathogens, we have previously reported fluctuating levels of pneumococcal resistance to penicillin in Malawi, ranging from 9·0 to 18·0%,^18^ as was also reported in other African settings, such as Senegal.^27^ Few studies in sub-Saharan Africa have described the prevalence of meticillin-resistant *Staphylococcus aureus* (MRSA) and although MRSA has spread in Malawi, its prevalence remains unknown.^28^

Surveillance data about both long-term bacteraemia and antimicrobial resistance are scarce in sub-Saharan Africa. We used our comprehensive sentinel surveillance dataset to describe longitudinal trends in bloodstream infection and antimicrobial resistance over 19 years at a large teaching hospital in Blantyre, Malawi, with particular focus on prevalence of ESBL and fluoroquinolone resistance among Gram-negative pathogens, and the emergence of MRSA.

## Methods

### Study design and procedures

In this surveillance study, we analysed blood cultures that were routinely taken from adult and paediatric patients with fever or suspicion of sepsis admitted to QECH, Blantyre, Malawi between 1998 and 2016.

The Malawi-Liverpool-Wellcome Trust Clinical Research Programme has provided routine, quality controlled, diagnostic blood culture service for febrile adult and paediatric medical patients admitted to QECH since 1998. A recommended 7–10 mL of blood were taken for culture under aseptic conditions from all adult patients admitted to the hospital with fever (axillary temperature >37·5°C) or clinical suspicion of sepsis, severe sepsis, or septic shock.^29^ Sepsis, severe sepsis, or septic shock were suspected in patients with tachycardia (≥90 beats per minute), hypotension (systolic blood pressure <90 mm Hg), tachypnoea (respiratory rate >20 per minute), or delirium. 3–10 mL of blood was taken from children with non-focal febrile illness who tested negative for malaria, who were severely ill with suspected sepsis, or who failed initial malaria treatment and remained febrile.^18^ In this busy hospital, afebrile patients were unlikely to have blood sampled for culture unless critically ill with suspected sepsis. If patients were critically ill and sample for culture was taken, the patients were not excluded from analysis.

Since 2000, blood was inoculated into a single aerobic bottle using the automated BacT/ALERT system (bioMérieux, France)^6^ before which, manual culture was used.^30^ Enterobacteriaceae and oxidase-positive Gram-negative bacilli were identified by API (BioMérieux, France), staphylococci by tube coagulase, β-haemolytic streptococci by Lancefield antigen testing, and salmonella by serotyping according to the White-Kauffmann-Le Minor scheme by the polyvalent O & H, O4, O9, Hd, Hg, Hi, Hm, and Vi antisera (Pro-Lab Diagnostics, UK). The identification of a sample of isolates as *Salmonella enterica* serotype Typhimurium was subsequently substantiated by whole genome sequencing and multi-locus sequence typing. *Haemophilus influenzae* was typed using type B antisera. Bacteria that form part of the normal skin or oral flora, including diphtheroids, bacilli, micrococci, coagulase-negative staphylococci, and α-haemolytic streptococci (other than *S pneumoniae*), were considered to be contaminants.^31^

Antimicrobial susceptibility tests were done by the disc diffusion method following the British Society of Antimicrobial Chemotherapy (BSAC) methods and breakpoints. Testing was in most cases limited to one plate containing six discs, and the choice of agent varied depending on the range of antimicrobials available to clinicians. Standard operating procedures are included in the appendix. Bacteria were defined as being resistant to Malawian first-line drugs (hereafter, RFL) if they were resistant to the three first-line antimicrobials commonly used in Malawi: amoxicillin, co-trimoxazole, and chloramphenicol for Gram-negative isolates; or penicillin, co-trimoxazole, and chloramphenicol for Gram-positive isolates. Isolates were considered multidrug resistant if they were resistant to three or more classes of antimicrobials to which reference strains are susceptible.^32^ Gram-negative isolates have been screened for ESBL-producing status using a cefpodoxime disc since 2007. Before then, ESBL was inferred on the basis of resistance to ceftriaxone. Meticillin resistance in *S aureus* was inferred by cefoxitin resistance, which replaced oxacillin resistance testing in 2010.

### Statistical analysis

We reported the prevalence of blood culture collection and causes of bloodstream infection using frequency distributions. Minimum annual incidence rates were expressed as incidence per 100 000 age-specific person-years and estimated by dividing the number of bloodstream infections by mid-year population and multiplying by 100 000. We used the Cochran-Armitage test for trend to examine trends in rates of resistance to antimicrobials over time, and negative binomial regression to examine trends in incidence of bloodstream infection over time. We obtained age-stratified population estimates for urban Blantyre for 1998–2007 from the 1998 National Population Projections and for 2008–16 from the 2008 National Population Projections by the National Statistical Office. Statistical analyses were done using R version 3.1.2 for MacOS. Blood culture surveillance at QECH was approved by the College of Medicine Research Ethics Committee (COMREC) of the University of Malawi, approval number P.08/14/1614.

### Role of the funding source

The sponsors of the study had no role in study design, data collection, data analysis, data interpretation, or writing of the report. The corresponding author had full access to all the data in the study and had final responsibility for the decision to submit the paper for publication.

## Results

**Figure 1:**
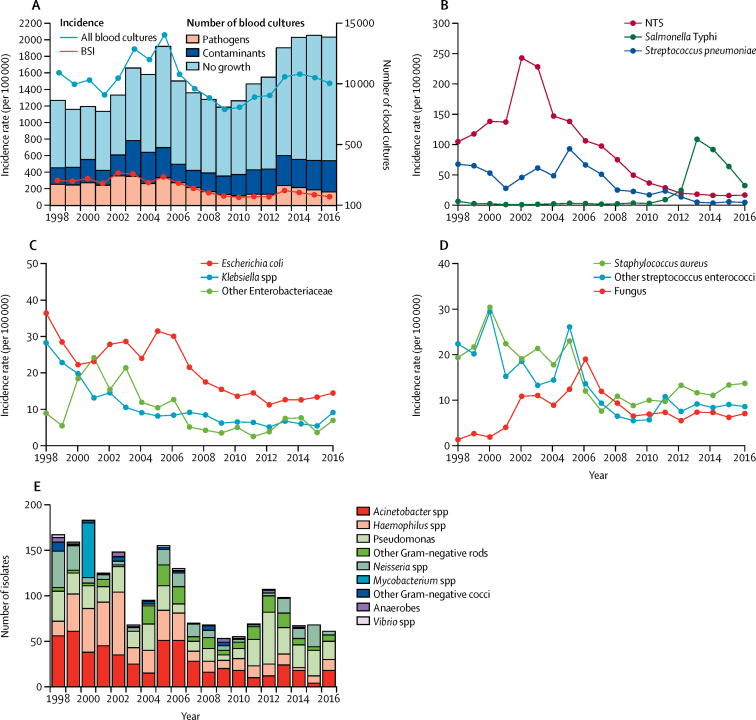
Trends in bloodstream infection, 1998–2016 (A) Annual frequency of blood culture sampling, and pathogen and contaminant isolation, plus estimated minimum incidence rates of bloodstream infection. (B) Estimated minimum incidence of pathogens isolated at high frequency (≥300/year). (C) Estimated minimum incidence of Gram-negative pathogens isolated at intermediate frequency (50–299/year). (D) Estimated minimum incidence of Gram-positive pathogens isolated at intermediate frequency (50–299/year). (E) Pathogens isolated at low frequency (<50/year). BSI=bloodstream infections. NTS=non-typhoidal salmonella.

Between Jan 1, 1998, and Dec 31, 2016, 194 539 blood cultures were collected from adults (79 095 [40·7%]) and children (115 444 [59·3%]) presenting to QECH. The absolute number of blood cultures collected per year fluctuated during the surveillance period (figure 1A). The ratio of blood cultures to population size increased up to 2005, before falling thereafter (figure 1A). 29 183 (15·0%) blood cultures yielded pathogens (table 1), a further 36 763 (18·9%) yielded contaminants (figure 1A; appendix) and there was no growth from 128 593 (66·1%) blood cultures (figure 1A). The estimated incidence rate of bloodstream infection declined substantially during the study period from 327·1/100 000 in 1998, to 120·2/100 000 in 2016 (figure 1A; p<0·0001). The pathogen profiles in children and adults are shown in the appendix.

**Table 1:** Prevalence of significant pathogens, 1998–2016

	**1998–2001**	**2002–05**	**2006–09**	**2010–13**	**2014–16**	**Total**
*Acinetobacter* spp	200 (3·0%)	126 (1·0%)	115 (2·0%)	64 (1·5%)	40 (1·0%)	545 (1·9%)
Anaerobes	7 (<0·1%)	8 (<0·1%)	7 (<0·1%)	6 (0·1%)	2 (0·1%)	30 (0·1%)
*Citrobacter* spp	69 (1·0%)	75 (0·8%)	20 (0·4%)	9 (0·2%)	12 (0·3%)	185 (0·6%)
*Escherichia coli*	592 (9·0%)	661 (7·0%)	552 (10·0%)	398 (9·3%)	357 (9·3%)	2560 (8·8%)
*Enterococcus faecalis*	48 (0·7%)	57 (0·6%)	61 (1·1%)	27 (0·6%)	27 (0·7%)	220 (0·8%)
*Edwardsiella* spp	0	2 (<0·1%)	0	0	0	2 (<0·1%)
Enterobacter	93 (1·3%)	173 (2·0%)	88 (1·6%)	74 (1·7%)	89 (2·3%)	517 (1·8%)
*Enterococcus* spp	14 (0·2%)	2 (<0·1%)	2 (<0·1%)	64 (1·5%)	69 (1·8%)	151 (0·5%)
*Escherichia* spp	0	3 (<0·1%)	3 (0·1%)	0	0	6 (<0·1%)
*Flavobacteria* spp	2 (<0·1%)	2 (<0·1%)	2 (<0·1%)	0	0	6 (<0·1%)
*Haemophilus influenzae* type b	112 (1·6%)	92 (1·0%)	26 (0·5%)	30 (0·7%)	15 (0·4%)	275 (0·9%)
*Haemophilus* spp	41 (0·6%)	53 (0·6%)	36 (0·7%)	21 (0·5%)	8 (0·2%)	434 (0·5%)
*Hafnia* spp	3 (<0·1%)	2 (<0·1%)	1 (<0·1%)	0	0	6 (<0·1%)
*Klebsiella* spp	449 (7·0%)	248 (3·0%)	211 (4·0%)	190 (4·4%)	183 (4·8%)	1281 (4·4%)
*Kluyvera* spp	2 (<0·1%)	1 (<0·1%)	2 (<0·1%)	2 (<0·1%)	0	7 (<0·1%)
Morganella morganii	0	3 (<0·1%)	4 (0·1%)	11 (0·3%)	1 (<0·1%)	19 (0·1%)
Mycobacterium	60 (1·0%)	0	0	0	0	60 (0·2%)
Neisseria	78 (1·0%)	27 (<0·1%)	42 (1·0%)	34 (0·8%)	39 (1·0%)	220 (0·8%)
Other Gram-negative cocci	15 (<0·1%)	11 (<0·1%)	11 (0·2%)	1 (<0·1%)	0	38 (0·1%)
Other Gram-negative rods	18 (<0·1%)	45 (1·0%)	41 (1·0%)	55 (1·3%)	7 (0·2%)	166 (0·6%)
Non-typhoidal salmonella	2685 (38·9%)	4432 (50·1%)	2141 (40·2%)	782 (18·3%)	433 (11·3%)	10 473 (35·9%)
*Streptococcus* spp	115 (1·7%)	113 (1·3%)	55 (1·0%)	46 (1·1%)	57 (1·5%)	386 (1·3%)
*Pantoea* spp	0	0	0	11 (0·3%)	9 (0·2%)	20 (0·1%)
*Proteus* spp	47 (0·7%)	9 (0·1%)	12 (0·2%)	7 (0·2%)	18 (0·5%)	93 (0·3%)
Pseudomonas	98 (1·0%)	102 (1·0%)	41 (1·0%)	126 (2·9%)	75 (2·0%)	442 (1·5%)
*Raoultella* spp	0	0	0	3 (0·1%)	8 (0·2%)	11 (<0·1%)
*Streptococcus agalactiae*	173 (2·5%)	155 (1·8%)	40 (0·8%)	70 (1·3%)	17 (0·4%)	455 (1·6%)
*Staphylococcus aureus*	505 (7·0%)	480 (5·0%)	258 (5·0%)	344 (8·0%)	338 (8·8%)	1925 (6·6%)
*Streptococcus pneumoniae*	1139 (17·0%)	1476 (17·0%)	1072 (20·0%)	448 (10·5%)	123 (3·2%)	4258 (14·6%)
Group A streptococcus	117 (1·7%)	102 (1·2%)	69 (1·3%)	59 (1·4%)	50 (1·3%)	397 (1·4%)
*Salmonella* Typhi	67 (1·0%)	49 (1·0%)	70 (1·0%)	1168 (27·3%)	1643 (43·0%)	2997 (10·3%)
*Serratia* spp	92 (1·3%)	66 (0·7%)	15 (0·3%)	21 (0·5%)	17 (0·4%)	211 (0·7%)
*Shigella* spp	1 (<0·1%)	9 (0·1%)	18 (0·3%)	6 (0·1%)	6 (0·2%)	40 (0·1%)
*Vibrio* spp	5 (<0·1%)	2 (<0·1%)	2 (<0·1%)	3 (0·1%)	0	12 (0·0)
Yersinia	2 (<0·1%)	2 (<0·1%)	1 (<0·1%)	2 (<0·1%)	0	7 (0·0)
*Candida* spp	4 (<0·1%)	6 (0·1%)	4 (0·1%)	13 (0·3%)	13 (0·3%)	40 (0·1%)
*Cryptococcus* spp	50 (1·0%)	249 (2·8%)	300 (5·6%)	195 (4·6%)	169 (4·4%)	963 (3·3%)
Total	6903	8843	5322	4290	3825	29 183

Data are n (%).

Trends in salmonella bloodstream infection revealed epidemics of non-typhoidal salmonella that peaked in 2002, and an epidemic of typhoid fever was described in detail up to 2014,^6^ but are included in table 1 and figure 1 to place other causes of bloodstream infections in context. Our analysis further shows that the *S* Typhi epidemic has been declining since its peak in 2013 (figure 1B). Of 29 183 bloodstream infections, 2560 (8·8%) were *E coli*, 1281 (4·4%) were *Klebsiella* spp, and 1130 (3·9%) were isolates of other species of Enterobacteriaceae. The most common other Enterobacteriaceae were *Enterobacter* spp (n=517), *Serratia* spp (n=211), *Citrobacter* spp (n=185), *Proteus* spp (n=93), and *Shigella* spp (n=40; table 1). Incidence of bloodstream infection attributable to these pathogens significantly declined over time (figure 1B, 1C; p<0·0001).

Other Gram-negative causes of bloodstream infection included *Acinetobacter* spp (n=543), *Haemophilus* spp (n=434), *Pseudomonas* spp (n=442), *Neisseria* spp (n=210), other anaerobes (30), and *Vibrio* spp (n=12). These together were responsible for 1671 (5·7%) of 29 183 positive blood cultures (figure 1E).

Trends in *S pneumoniae* bloodstream infection have been described for 2000–09,^18^ whereas this dataset spans 1998–2016. In this longer period, 4258 (14·6%) of 29 183 bloodstream infections were *S pneumoniae* isolates. The annual incidence of bloodstream infection due to *S pneumoniae* decreased significantly (figure 1B; p<0·0001).

*S aureus* was the second most common Gram-positive cause of bloodstream infection with 1923 (6·6%) of 29 183 isolates during the study period. Isolation of *S aureus* fluctuated throughout the study period (figure 1D), but the incidence of *S aureus* bloodstream infection declined significantly overall (p<0·0001).

1193 (4·5%) of 29 183 bloodstream infections were β-haemolytic *Streptococcus* spp, (including 397 [1·4%] group A and 477 [1·6%] group B) and 320 (1·0%) were *Enterococcus* spp (including 220 [0·8%] *E faecalis,* 76 [0·3%] *E faecium,* and 42 [0·1%] other *Enterococcus* spp). Whereas the incidence of β-haemolytic streptococci declined (p=0·0005) during surveillance, there was no significant change in enterococcal bloodstream infection (p=0·4900). We isolated yeast in 1003 (34%) of 29 183 samples (table 1), including 963 isolates of *Cryptococcus neoformans* and 40 of *Candida* spp. After consistently increasing between 1998 and 2006, the incidence of fungus bloodstream infection has declined since 2006 (figure 1D).

Of the 29 183 culture-confirmed bloodstream infections, the age of the patient was known for 23 219 (79·6%) samples. 13 002 (56·0%) patients with known age were children (<16 years) and 10 217 (44·0%) were adults (≥16 years old). 10 059 (77·4%) children were younger than 5 years. Most bacterial species had a bimodal age distribution, affecting mostly children under 5 years of age and adults aged 20–45 years (appendix). The only exception was *S* Typhi, which was most common in children younger than 10 years.^6^ Cryptococcal bloodstream infection was most common in adults aged 20–45 years, but uncommon in children (appendix).

**Figure 2:**
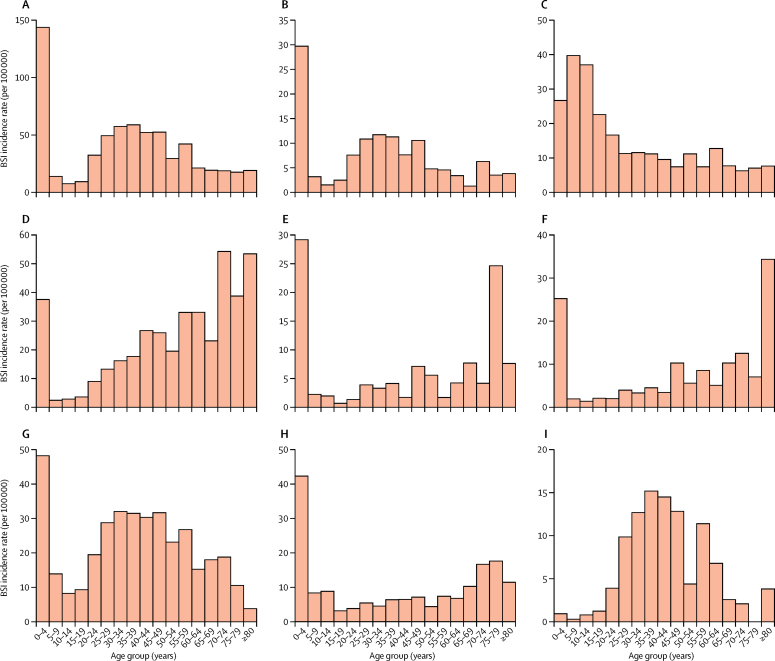
Estimated minimum incidence rates of bloodstream infection stratified by age (A) *Salmonella* Typhimurium. (B) *S* Enteritidis. (C) *S* Typhi. (D) *Escherichia coli.* (E) *Klebsiella* spp. (F) Other Enterobacteriaceae. (G) *Streptococcus pneumoniae.* (H) *Staphylococcus aureus.* (I) Yeast. BSI=bloodstream infections.

When the aggregate data were adjusted to produce minimum incidence estimates stratified by age, a subtly different picture emerged. The incidence rates for *E coli* bloodstream infection were greatest in patients aged 70 years or older (up to 54·3/100 000 per year) followed by children younger than 5 years (37·5/100 000 per year); and for klebsiella bloodstream infection, incidence rates were highest in children younger than 5 years (29·2/100 000 per year) followed by people aged 75–80 years (24·6/100 000 per year). For *S* Typhi, incidence rates were greatest in children between 5 and 10 years and for all other bacterial pathogens they were greatest in those younger than 5 years of age (figure 2).

**Figure 3:**
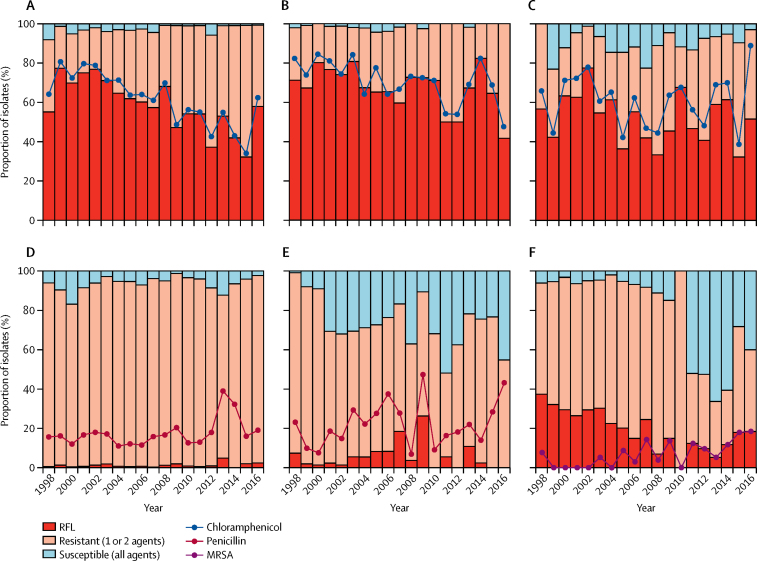
Trends in proportions of isolates resistant to Malawian first-line antimicrobials (A) *Escherichia coli.* (B) *Klebsiella* spp. (C) Other Enterobacteriaceae. (D) *S pneumoniae.* (E) *Staphylococcus aureus.* (F) Other *Streptococcus and Enterococcus* spp. First-line antimicrobials include chloramphenicol and co-trimoxazole, plus ampicillin for Gram-negative pathogens and penicillin for Gram-positive pathogens. RFL=resistant to all first-line antimicrobials.

27 249 (96·7%) of 28 180 confirmed bacterial bloodstream infection isolates were tested for susceptibility to at least one antimicrobial agent and 25 752 (91·4%) of 28 180 bloodstream infection isolates were tested for susceptibility to at least three antimicrobial agents. 13 343 (52·2%) of 25 572 isolates tested for susceptibility to at least three agents were RFL, 10 316 (40·3%) of 25 572 were resistant to one or two first-line agents, and 2093 (8·2%) of 25 572 were susceptible to all three first-line agents (figure 3). Overall, proportions of RFL isolates increased during the surveillance period (appendix; p<0.0001). RFL was markedly more common among Gram-negative isolates (12 902 [68·3%] of 18 887) than Gram-positive isolates (441 [6·6%] of 6685). 6129 (91·7%) of 6685 Gram-positive isolates were susceptible to either penicillin or chloramphenicol.

By contrast with the overall trends in RFL isolates, the proportion of *E coli* isolates that were RFL declined substantially during the study period (p<0·0001), primarily due to a decline in chloramphenicol resistance (figure 3A; p<0·0001). Chloramphenicol resistance also declined in *Klebsiella* spp (p<0·0001), but increased in salmonella (appendix), while no significant trend was detected in other members of the Enterobacteriaceae (figure 3C; p=0·2203).

**Figure 4:**
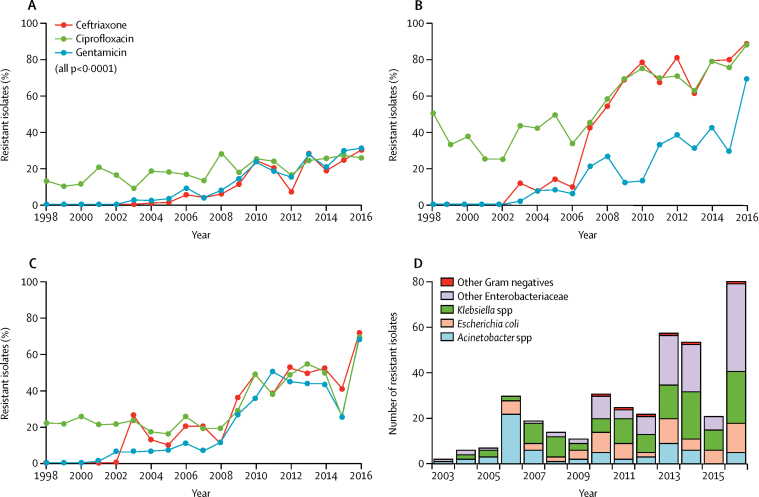
Trends in resistance to second-line antimicrobial agents ciprofloxacin, ceftriaxone, and gentamicin (A) *Escherischia coli.* (B) *Klebsiella spp*. (C) Other Enterobacteriaceae.(D) Trend in number of isolates resistant to all six commonly used antimicrobial agents in Malawi (ampicillin, chloramphenicol, cotrimoxazole, ceftriaxone, ciprofloxacin, and gentamicin).

ESBL production was first detected in *E coli* in 2004 and in *Klebsiella* spp and other Enterobacteriaceae in 2003. Both frequency and incidence of ESBL-producing isolates have since increased markedly in all non-salmonella Enterobacteriaceae (figure 4; appendix). In addition to the Enterobacteriaceae, most (168 [61·3%] of 274) *Acinetobacter* spp isolates were ESBL producers and there was also an increasing trend of ESBL *Acinetobacter* spp isolates (appendix). Ceftazidime was not widely available, therefore pseudomonas isolates were not routinely tested; however, seven of nine pseudomonas isolates tested were resistant to ceftazidime.

Ciprofloxacin resistance was first detected in Blantyre in acinetobacter isolates (four [2·3%] of 43) in 2001 and in *E coli* (four [2·5%] of 22) and klebsiella (one [1·7%] of 60) isolates in 2003. As with ESBL resistance, we observed an increasing trend in both the proportion and rate of non-salmonella Enterobacteriaceae with resistance to fluoroquinolones (figure 4). 105 (26·7%) of 393 acinetobacter isolates and 55 (12·8%) of 344 pseudomonas isolates were resistant to ciprofloxacin.

We detected gentamicin resistance in 462 (18·2%) of 2536 *E coli*, 565 (51·9%) of 1265 *Klebsiella* spp, and 320 (29·7%) of 1076 other Enterobacteriaceae, with substantial increases in proportions of resistant isolates over time (figure 4). 210 (40·1%) of 524 acinetobacter and 140 (33·6%) of 417 pseudomonas isolates were also resistant to gentamicin.

Only 37 (0·9%) of 3049 *S pneumoniae* isolates were resistant to all Malawian first-line agents (figure 3D). Resistance to co-trimoxazole was common (3780 [92·5%] of 4087 isolates). By contrast, only 610 (15·1%) of 4043 isolates were resistant or intermediate resistant to penicillin (falling to 551 [13·6%] of 4043 when intermediate resistance was excluded). The overall trend in penicillin resistant isolates was not significant (p=0·2300) but a marked rise occurred after the introduction of the pneumococcal conjugate vaccine (PCV13) in 2011 (figure 3D; p<0·0001). 1074 (26·1%) of 4111 *S pneumoniae* isolates were resistant to chloramphenicol. Of isolates tested for susceptibility to both chloramphenicol and penicillin, 3971 (99·0%) of 4013 were susceptible to at least one of the two antimicrobial agents. 2200 (53·9%) of 4080 *S pneumoniae* isolates were resistant to tetracycline and 92 (2·2%) of 4107 *S pneumoniae* isolates were resistant to the macrolide erythromycin.

1151 (79·0%) of 1457 *S aureus* isolates were resistant to penicillin, 828 (43·7%) of 1895 were resistant to co-trimoxazole, and 452 (23·8%) of 1898 to chloramphenicol. 107 (9·6%) of 1118 *S aureus* isolates were MRSA, which was first detected in 1998, but was not regularly isolated until 2005 (figure 3E). Only 20 of 1681 *S aureus* isolates were tested for susceptibility to ciprofloxacin and none was resistant. 206 (11·5%) of 1790 isolates tested were resistant to gentamicin.

855 (70·5%) of 1213 β-haemolytic streptococci isolates and 229 (70·5%) of 325 enterococci isolates were resistant to co-trimoxazole, whereas 176 (15·3%) of 1149 streptococci isolates and 108 (61·4%) of 176 *Enterococci* spp were resistant to penicillin and ampicillin. 208 (17·1%) of 1143 streptococci and 198 (60·7%) of 326 enterococci were resistant to chloramphenicol. Among group A streptococci, 14 (3·7%) of 382 were resistant to penicillin; however, these isolates were not speciated and we are therefore unable to specifically identify the *Streptococcus pyogenes* isolates within this group.

Most *Salmonella* spp, *E coli,* klebsiella, and indeed all other Enterobacteriaceae were multidrug resistant, as were a substantial proportion of *S pneumoniae,* other streptococci, and enterococci (table 2). Trends in multidrug-resistant isolates were increasing in *Klebsiella* spp (p<0·0001) and in other *Streptococcus* spp and *Enterococcus* spp (p<0·0001). We detected a decline in multidrug resistance (p<0·0001) in *E coli* isolates (table 2).

**Table 2:** Trends in multidrug-resistant bacterial bloodstream infection pathogens in Blantyre, 1998–2016

	***Escherichia coli***	***Klebsiella* spp**	**Enterobacteriaceae**	***Streptococcus* pneumoniae**	***Enterococcus* spp**	***Streptococcus* spp**
1998	111/185 (60%)	66/146 (45%)	22/30 (73%)	85/335 (25%)	18/26 (69·%)	18/90 (20%)
1999	118/150 (79%)	31/113 (27%)	12/26 (46%)	98/313 (31%)	8/13 (62%)	15/93 (16%)
2000	83/116 (72%)	38/101 (38%)	60/90 (67%)	80/238 (34%)	3/8 (38%)	21/143 (15%)
2001	96/124 (77%)	17/73 (23%)	87/131 (66%)	41/154 (27%)	10/15 (67%)	13/70 (19%)
2002	113/147 (77%)	20/81 (25%)	64/80 (80%)	75/231 (33%)	2/7 (29%)	8/72 (11%)
2003	110/152 (72%)	24/52 (46%)	67/108 (62%)	116/316 (37%)	2/3 (67%)	13/74 (18%)
2004	92/133 (69%)	21/46 (46%)	39/62 (63%)	83/265 (31%)	5/7 (71%)	22/79 (28%)
2005	121/181 (67%)	22/46 (48%)	24/55 (44%)	158/494 (32%)	32/41 (78%)	28/114 (25%)
2006	84/186 (45%)	18/52 (35%)	45/76 (59%)	110/413 (27%)	6/13 (46%)	15/70 (21%)
2007	81/136 (60%)	27/57 (47%)	15/31 (48%)	105/320 (33%)	9/14 (64%)	10/46 (22%)
2008	81/116 (70%)	36/55 (66%)	10/27 (37%)	54/160 (34%)	12/14 (86%)	6/29 (21%)
2009	60/106 (57%)	29/40 (73%)	12/22 (55%)	47/151 (31%)	12/17 (71%)	4/16 (25%)
2010	64/96 (67%)	34/45 (76%)	25/34 (74%)	34/119 (29%)	10/13 (77%)	13/28 (46%)
2011	76/109 (70%)	35/48 (73%)	10/15 (67%)	71/177 (40%)	6/7 (86%)	28/71 (39%)
2012	42/86 (49%)	32/38 (84%)	17/27 (63%)	30/106 (28%)	15/16 (94%)	7/41 (17%)
2013	74/100 (74%)	33/55 (60%)	42/61 (69%)	12/41 (29%)	15/17 (88%)	25/58 (43%)
2014	63/105 (60%)	41/51 (80%)	40/57 (70%)	12/31 (39%)	15/18 (83%)	16/53 (30%)
2015	65/118 (55%)	39/48 (81%)	10/33 (30%)	25/50 (50%)	29/34 (85%)	19/46 (40%)
2016	92/133 (69%)	77/84 (92%)	51/65 (79%)	9/42 (21%)	37/48 (77%)	8/31 (26%)
Overall	1626/2479 (66%)	640/1231 (52%)	652/932 (63%)	1245/3956 (32%)	246/331 (74%)	289/1224 (24%)
p value	<0·0001*	<0·0001†	0·747	0·148	<0·0001†	<0·0001†

† Increasing trend. Isolates are considered multidrug resistant when resistant to at least three antimicrobial classes.

* Decreasing trend.

381 Gram-negative isolates were resistant to all agents tested, including amoxicillin, co-trimoxazole, chloramphenicol, gentamicin, ciprofloxacin, and ceftriaxone, rendering them locally untreatable. Of these 381 isolates, 121 (31·8%) were *Klebsiella* spp, 68 (17·8%) were *E coli*, 119 (26·0%) were various other Enterobacteriaceae, and 119 (31·2%) were acinetobacter (figure 4D). The number of isolates expressing resistance to all the six agents was 81 (7·3%) of 1106 bloodstream infections in 2016, increased from two (<0·1%) of 2372 bloodstream isolates in 2003 (figure 4D).

## Discussion

Long-term sentinel surveillance in Malawi shows a marked decline in the incidence of bloodstream infection caused by all pathogens from 1998 to 2016. However, concurrent with the declining incidence has been an increase in the prevalence of antimicrobial resistance—including resistance to reserve antimicrobials. These changes occurred against a background of improvements in food security, malaria control interventions, and highly successful roll-out of antiretroviral therapy. Following the emergence of widespread resistance to commonly available first-line antimicrobial agents, cephalosporins and fluoroquinolones have become the drugs of choice for treatment of severe bacterial infections in sub-Saharan Africa.^33^ The emergence and spread of ESBL-resistance and fluoroquinolone resistance is therefore a major concern in this setting. The prevalences of ESBL resistance and fluoroquinolone resistance in *E coli* and klebsiella spp were high, for Africa and when considered worldwide.^34^, ^35^, ^36^ In other settings, ESBL and fluoroquinolone-resistant pathogens are more common in hospital-acquired infections than in community-acquired infections; this study deals with only community-acquired bacteraemia. Therefore, the findings in this study might underestimate the overall rates of ESBL resistance in this setting.^37^, ^38^

Our findings suggest that the incidence of *E coli* and klebsiella bloodstream infections is highest in elderly patients, consistent with global data.^39^ The Malawi National Statistical Office estimates that life expectancy in Blantyre will increase from about 55 years in 2007, to about 70 years by 2030. Increased life expectancy might increase the pool of people at risk of *E coli* and *Klebsiella* bloodstream infections, with the attendant increasing risk of drug resistance.

Resistance to first-line antimicrobials has fluctuated. In some Enterobacteriaceae such as *E coli* and *Klebsiella* spp, RFL rates have begun to decline, primarily due to less chloramphenicol resistance. However, the molecular determinants of this observation in *E coli* and *Klebsiella* spp are unknown. This partial re-emergence of chloramphenicol susceptibility is not sufficiently great to permit its reintroduction as an empirical treatment for sepsis in Blantyre because of widespread resistance among salmonella, the dominant Gram-negative pathogens.

Chloramphenicol and penicillin have been commonly used in combination for the empirical management of sepsis in Malawi for many years.^18^ 99·0% of the *S pneumoniae* isolates are still susceptible to this combination despite its wide usage. However, penicillin resistance has started to increase since the introduction of PCV13 in 2011. PCV13 introduction has been associated with a general decline in penicillin-resistant *S pneumoniae* in South Africa.^40^ However, increasing prevalence of penicillin-resistant *S pneumoniae* serotype 19F and non-vaccine serotypes such as 19A and 15A have been reported following the introduction of the PCV7 and PCV13 outside sub-Saharan Africa.^41^, ^42^ The increase in the proportion *S pneumoniae* that are resistant to penicillin when *S pneumoniae* bloodstream infections are decreasing raises the possibility that there has been a change in serotype distribution following vaccine introduction as has been the case in the other settings.^41^, ^42^

We also describe the emergence of MRSA, although it remains an infrequent cause of bloodstream infection in Blantyre. The prevalence of MRSA among the *S aureus* isolates was similar to proportions from countries such as Mozambique and Zimbabwe, but much lower than those reported in South Africa.^43^, ^44^, ^45^ This difference might be because our cultures were taken from community admissions to medical wards; we have yet to study nosocomial infection in-depth in our setting. It might also be because, unlike in South Africa, medical devices that place patients at risk of MRSA bloodstream infection (such as central venous catheters) are rarely used in low-income countries such as Malawi. The sustained presence of MRSA as a low-level cause of bloodstream infection in Blantyre is of considerable concern. Its relative importance as a bloodstream infection pathogen could change greatly if surveillance expands to cover surgical patients or nosocomial infections or if medical practice changes.

This study has several limitations. The median length of stay for adult internal medicine inpatients was 5 days, and was shorter for children.^46^ Typically patients at QECH undergo blood culture on admission but it was uncommon for patients to have follow-up blood culture and it is therefore unlikely that our surveillance has captured much nosocomial infection. Community-acquired sepsis could have been missed if people died at home or were not referred to hospital, thus we consider our rates to be minimum estimates.

An antimicrobial susceptibility profile was described for 96·7% of the isolates and reflect British Society of Antimicrobial Chemotherapy guidelines at the time. Screening for ESBL was not introduced until 2003, and as such ESBL-producing pathogens might have been circulating undetected before then. ESBL screening by cefpodoxime disc testing was not introduced until 2007, consequently some isolates might have been falsely classified as ESBL-producing before 2007. Cefoxitin screening for MRSA replaced meticillin screening in 2010, although the small increase in sensitivity gained will have made minimal difference to the findings.

The overall decreases in bacterial bloodstream infection have been accompanied by a rise in antimicrobial resistance in all bacterial bloodstream infection pathogens at QECH, especially in Gram-negative organisms, and the emergence of meticillin resistance in *S aureus.* Ceftriaxone and ciprofloxacin have been essential for the management of bacterial bloodstream infection in a setting where human immunosuppression and bacterial multidrug resistance are common. The emergence of ESBL, fluoroquinolone, and gentamicin resistance and MRSA highlight the growing challenge of bloodstream infections that are impossible to treat in this resource-limited setting.
