# Supplementary material for: Trends in antimicrobial resistance in bloodstream infection isolates at a large urban hospital in Malawi (1998–2016): a surveillance study
Source: Lancet Infect Dis. 2017 Oct;17(10):1042–52. doi: 10.1016/S1473-3099(17)30394-8 (PMC5610140; doi:10.1016/S1473-3099(17)30394-8)
Supplement: Supplementary appendix [file mmc1.pdf]

THE LANCET Infectious Diseases

Supplementary webappendix

This webappendix formed part of the original submission and has been peer reviewed. We post it as supplied by the authors.

Supplement to: Musicha P, Cornick JE, Bar-Zeev N, et al. PTrends in antimicrobial resistance in bloodstream infection isolates at a large urban hospital in Malawi (1998–2016): a surveillance study. *Lancet Infect Dis* 2017; published online August 14. http://dx.doi.org/10.1016/S1473-3099(17)30394-8.

SUPPLEMENTARY APPENDIX

Trends in antimicrobial resistance in bloodstream infection isolates at a large urban hospital in Malawi (1998-2016): a surveillance study

Patrick Musicha (MSc), Jennifer E. Cornick (PhD), Naor Bar-Zeev (PhD), Neil French (PhD), Clemens Masesa (MSc), Brigitte Denis (MSc), Neil Kennedy (MMedSci), Jane Mallewa (MD), Melita A. Gordon (MD), Chisomo L. Msefula (PhD), Robert S. Heyderman (PhD), Dean B. Everett (PhD), Nicholas A. Feasey (PhD).

SUPPLEMENTARY FIGURES

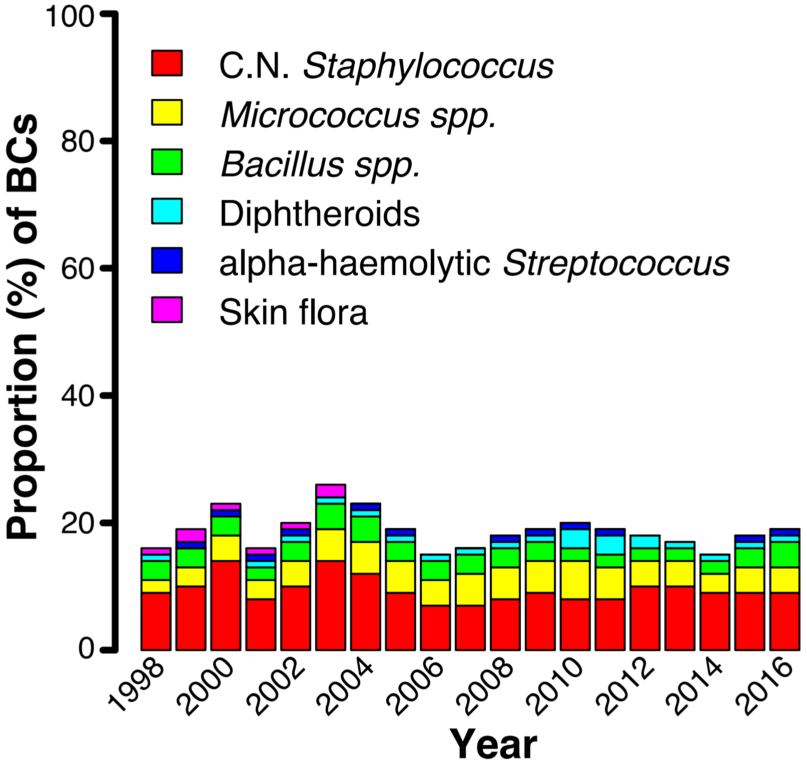

Supplementary Figure 1: Distribution of contaminants as proportions of all BCs by year of isolation.

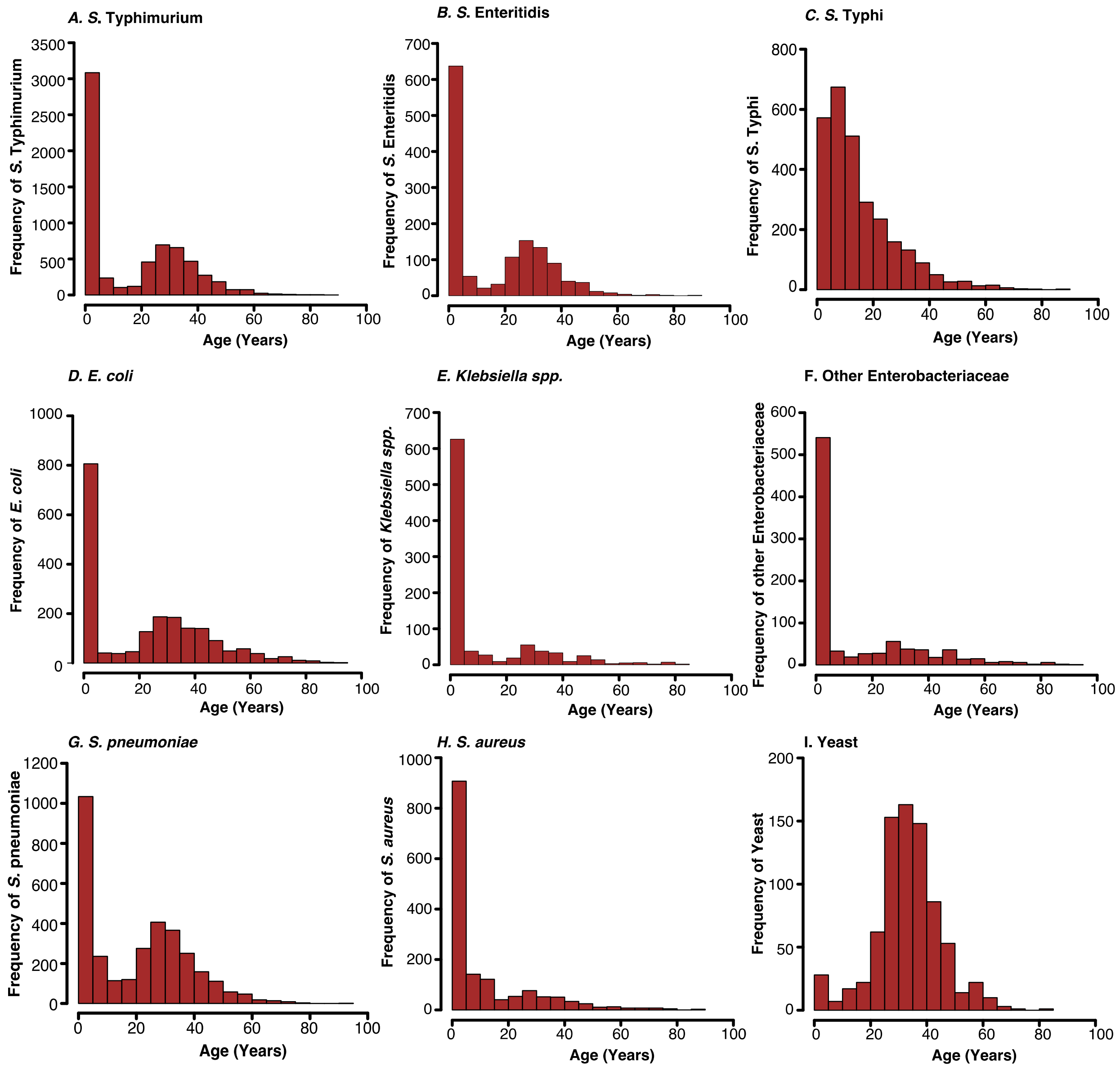

Supplementary Figure 2: Age distributions of patients presenting to QECH between 1998 and 2014 with confirmed BSI caused by (A) *S*. Typhimurium, (B) *S*. Enteritidis, (C) *S*. Typhi, (D) *E. coli*, (E) *Klebsiella spp*., (F) other Enterobacteriaceae, (G) *S. pneumoniae*, (H) *S. aureus* and (I) yeast.

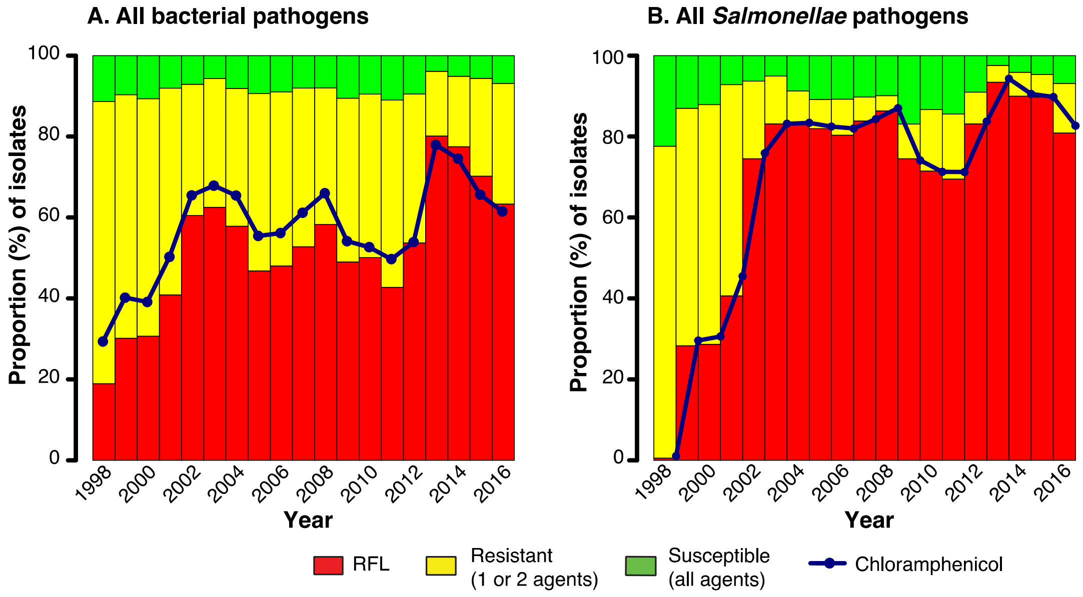

Supplementary Figure 3: Trends in resistance to first-line agents amongst in (A) all bacterial pathogens and (B). All *Salmonellae* isolates**.** As with Figure 3**,** first line antimicrobials include chloramphenicol and cotrimoxazole, plus ampicillin for gram negative pathogens and penicillin for gram positive pathogens

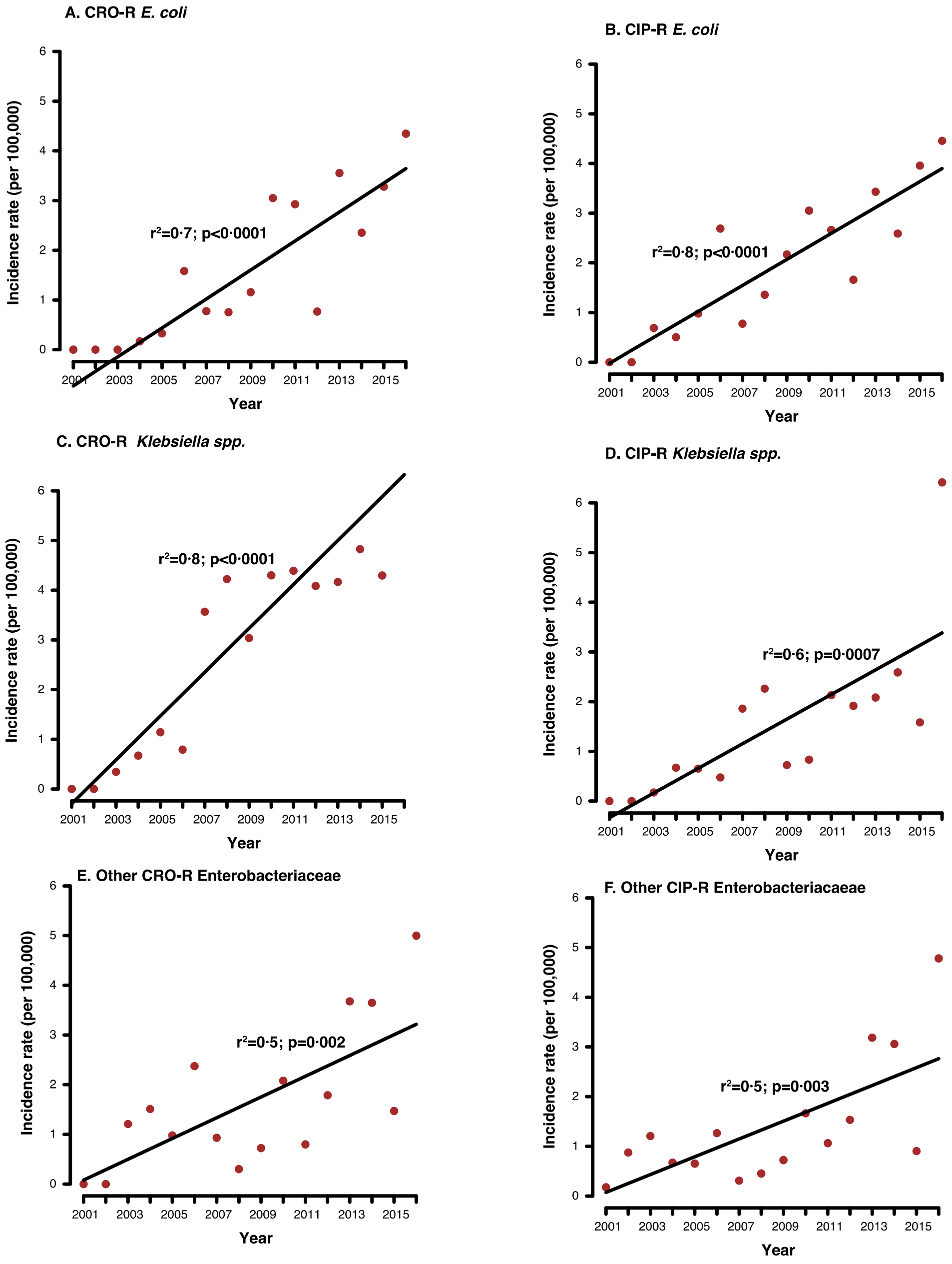

Supplementary Figure 4: Trends in annual incidence (per 100,000) of Enterobacteriaceae resistant to ceftriaxone (CRO-R) or ciprofloxacin (CIP-R). A. Annual incidence of CRO-R *E. coli*; B. Annual incidence of CIP-R *E. coli*; C. Annual incidence of CRO-R *Klebsiella spp*; Annual incidence of CIP-R *Klebsiella spp.* (E) Annual incidence of Other CRO-R Enterobacteriaceae; F. Annual incidence of other CIP-R Enterobacteriaceae. In For each panel, points represent observed annual incidence rates

while the black line graph represent annual incidence rates predicted by a linear regression model with the general form $\boldsymbol{y} = \boldsymbol{\beta_0} + \boldsymbol{\beta_1 x}$, where $\boldsymbol{y}$ is the incidence rate, $\boldsymbol{x}$ is the year isolation and $\boldsymbol{\beta_0}$ and $\boldsymbol{\beta_1}$ are constant coefficients.

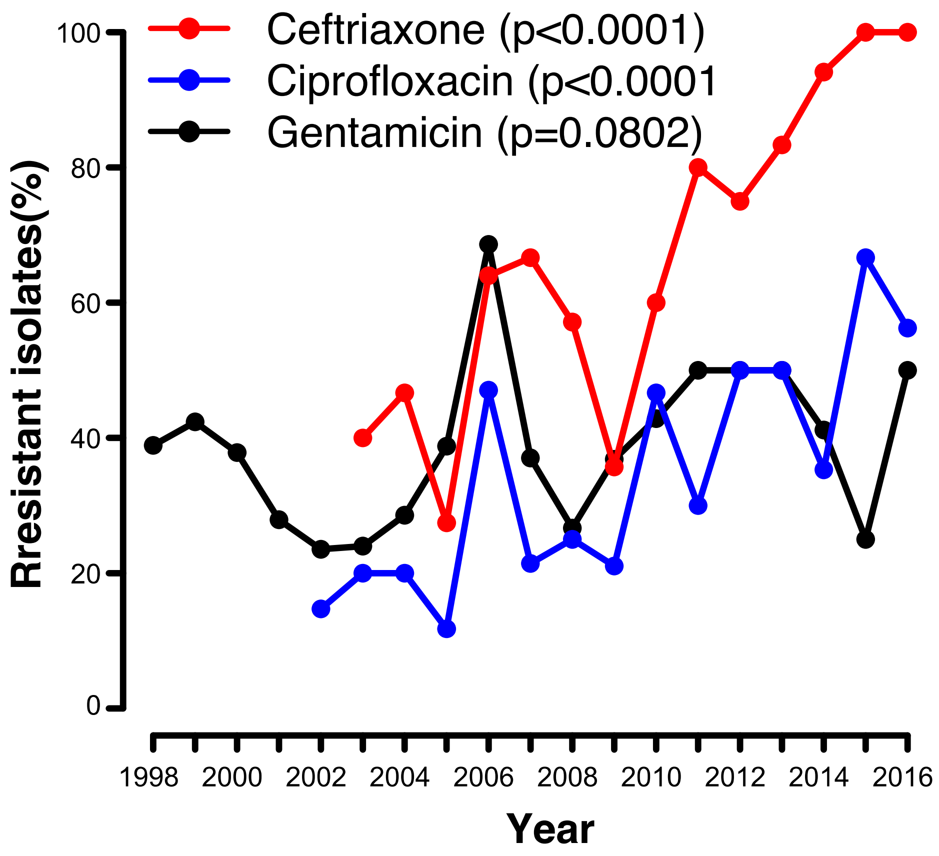

Supplementary Figure 5: Trends in proportions of *Acinetobacter spp.* isolates resistant to ceftriaxone, ciprofloxacin and gentamicin during 1998-2016.
